# Patterns of *Mycobacterium tuberculosis* genotypes and resistance mutations in patients with pulmonary multidrug-resistant tuberculosis in Sverdlovsk Oblast, Russia

**DOI:** 10.3389/ftubr.2025.1406536

**Published:** 2025-05-23

**Authors:** Tatiana Umpeleva, Elena Mazurina, Leonid Lavrenchuk, Sven G. Hinderaker, Einar Heldal, Andrei Mariandyshev, Diana Vakhrusheva, Irina Vasilieva

**Affiliations:** ^1^Department of Microbiology and Preclinical Research, National Medical Research Center for Phthisiopulmonology and Infection Disease, Yekaterinburg, Russia; ^2^Department of Global Public Health and Primary Care, University of Bergen, Bergen, Norway; ^3^LHL International, Oslo, Norway; ^4^Department Phthisiopulmonology, Northern State Medical University, Arkhangelsk, Russia; ^5^Research Department, Northern Arctic Federal University, Arkhangelsk, Russia; ^6^Administration, National Medical Research Center of Phthisiopulmonology and Infection Disease, Moscow, Russia

**Keywords:** tuberculosis, MDR, genotyping, mutation, TB-TEST

## Abstract

**Background:**

Nearly half of new tuberculosis patients in Sverdlovsk Oblast were diagnosed with multidrug-resistant tuberculosis (MDR-TB) and often exhibited fluoroquinolone resistance (FQ-R). This study aimed to (1) determine the number of MDR-TB patients who had *Mycobacterium tuberculosis* exhibiting the same genetic patterns (a unique combination of genotypes and mutations in genes associated with drug resistance) using the Russian microarray assay TB-TEST and (2) assess the correlation between these patterns and patient characteristics.

**Materials and methods:**

We analyzed 345 MDR-TB DNA samples from patients with pulmonary TB in Sverdlovsk Oblast between 2017 and 2020 using the TB-TEST. We assumed that isolates with unique patterns, which were seen in only one patient, indicated minimal transmission of *M. tuberculosis*, while the presence of more isolates with the same pattern suggested a more recent transmission. All patients were categorized into three groups to ensure that each group was approximately of the same size: Group 1 consisted of unique patterns; Group 2 (the low-frequency patterns group) included patterns shared by 2–6 patients; and Group 3, (the high-frequency patterns group, “dominant”) included patterns shared by 7–18 patients.

**Results:**

In total, 174 distinct genetic patterns were identified: Group 1 included unique patterns, accounting for 36.8%; Group 2 included low-frequency patterns, accounting for 31.0%; and Group 3 included high-frequency patterns, accounting for 32.2%. The Beijing B0/W148 genotype was found in 72.4% of cases, and it was significantly less frequent in patients with unique patterns (59.1% vs. 66.4% vs. 93.7%). Mutations in *gyrA/B* genes were found in 50.4% of all samples; however, these mutations were significantly more common in the group with unique patterns (73% vs. 43.9% and 30.6%). This suggests that the mutations in *gyrA/B* genes may have developed over the years because of inadequate chemotherapy regimens. Nevertheless, these mutations have not yet spread widely, possibly due to lower transmission potential or recent emergence. Patients with *M. tuberculosis*–positive sputum who had undergone treatment for more than 12 months demonstrated a significantly higher proportion of unique patterns and a higher rate of fluoroquinolone resistance.

**Conclusion:**

Patients with unique patterns were found to have MDR-TB. However, a higher proportion of *M. tuberculosis* with mutations in *gyrA/B* genes in the group with unique patterns may indicate reduced transmissibility.

## 1 Introduction

In Russia, the incidence rate of tuberculosis (TB) decreased by 5.7% per year during 2010–2019, reaching 50 per 100,000 in 2019. However, the proportion of patients with multidrug-resistant (MDR) TB (rifampicin + isoniazid resistance) increased to an estimated 35% in new cases in 2019 (1). The prevalence of MDR-TB varies significantly within the country, from 7.8% in the Chechen Republic to 66.7% in the Nenets Autonomous Okrug in 2020 (2). Sverdlovsk Oblast also reported a high prevalence, with 48% of MDR-TB in new patients in 2020. Furthermore, in 2019, it was found that 23.5% of MDR-TB patients in Russia exhibited resistance to both fluoroquinolone (FQ) (i.e., pre-extensively drug-resistant [pre-XDR]) and second-line injectable drugs (3). The proportion of MDR-TB cases has increased since 1990, likely due to inadequate treatment regimens, non-adherence to treatment, limited financing for TB support services (4), and increasing levels of HIV infection. In 2019, there were 16,453 (23.3%) TB patients who were HIV positive in Russia (3). Drug resistance can be acquired during ineffective TB treatment or transmitted from individuals who already have MDR-TB (5). The success of bacterial strains depends on many factors, including increased transmission because of delay in diagnosis (of TB and resistance), crowded living conditions, resistant strains when adequate regimens are not followed, and the biological properties of *Mycobacterium tuberculosis* (6), one of which may belong to a certain genetic line.

Beijing B0/W148 is recognized as an evolutionarily successful genetic clone of *M. tuberculosis*; it has been detected in several regions of Russia (7–9), including Sverdlovsk Oblast (10, 11), and is often associated with MDR-TB (10, 12, 13). However, the question remains regarding the contribution of different genotypes of *M. tuberculosis*, each with different combinations of drug-resistant mutations, to the MDR-TB epidemic. It is known that a higher number of resistant mutations is associated with *reduced* transmission rates, suggesting a global decrease in fitness on gradual accumulation of mutations conferring resistance (14). Many studies describe “compensatory mutations” that enable bacteria to restore their viability and adapt to competing conditions (15–17). Compensatory evolution allows resistant bacteria to survive even in the absence of antibiotic selection pressure (18, 19). Therefore, not all resistant bacterium variants have the same epidemiological significance, but those with additional genetic advantages tend to transmit more successfully than bacteria without these advantages.

Genotyping serves as a powerful tool for unraveling the epidemiological significance of diverse microbial genetic variants. Since 2014, the microarray-based TB-TEST (BIOCHIP-IMB, Russia) has been included in the Russian clinical recommendations (20), allowing the study of specific genotypes (21) endemic to Russia and mutations associated with drug resistance to rifampicin, isoniazid, ethambutol, aminoglycosides, and fluoroquinolones.

The specific objectives of this study, using data collected between 2017 and 2020 from MDR-TB patients at the Ural Research Institute of Phthisiopulmonology (UNIIF), were (1) to determine the number of MDR-TB patients who had *M. tuberculosis* with the same pattern (a unique combination of genotype and drug-resistant mutations) using the Russian microarray assay TB-TEST and (2) to assess the correlation between groups of these patterns and patient characteristics (gender, age, HIV/hepatitis co-infection, duration of treatment, and type of clinical material). We assumed that isolates with unique patterns indicated minimal transmission of *M. tuberculosis*, while more samples of *M. tuberculosis* sharing the same pattern suggested a more recent transmission.

## 2 Materials and methods

### 2.1 Setting

The population of Sverdlovsk Oblast was 4,310,681 in 2020, including 1,526,000 in Yekaterinburg, the administrative center of the Ural Federal District and Sverdlovsk Oblast. The notification rate of new TB cases per 100,000 population in Sverdlovsk Oblast decreased from 101 in 2003 to 52 in 2020. The proportion of MDR-TB among new cases gradually increased from 4% in 2003 to a very high but stable level of 47% in 2017 and 48% in 2020. HIV infection rates among TB patients in Sverdlovsk Oblast increased from 5% in 2006 to 40% in 2020 (3, 23). In 2018, among newly diagnosed TB patients with resistance to at least rifampicin, the treatment outcomes were as follows: 62% achieved successful treatment, 12% experienced failure, 14% resulted in death, 7.4% had treatment interruptions, and 4.6% continued treatment (22).

### 2.2 Study population

The UNIIF, a branch of the National Medical Research Center of Phthisiopulmonology and Infection Disease (NMRC), is located in Yekaterinburg. The institute provides advanced medical care to TB patients from different regions of Russia, with 60%−70% of patients coming from Sverdlovsk Oblast. The main reasons for hospitalization include diagnosis (particularly for complex cases where *M. tuberculosis* is absent in sputum), surgical treatment of TB (pulmonary resections and thoracoplasties), and treatment for other lung pathologies and MDR-TB. The lack of clinical and radiological improvement, despite receiving appropriate chemotherapy for TB, indicates the need for hospitalization in our clinic for further chemotherapy or surgical intervention. HIV-positive TB patients are admitted to the UNIIF only if their CD4 count is above 400 cells/ml; patients with lower CD4 counts are usually referred to specialized AIDS-TB hospitals in the Sverdlovsk region.

In this study, we included only patients diagnosed with pulmonary TB who were residing in Sverdlovsk Oblast and receiving treatment at the UNIIF–NMRC. The samples were processed in the microbiology laboratory and underwent polymerase chain reaction (PCR) testing between January 2017 and December 2020, with corresponding TB-TEST results available. We included both previously untreated TB patients (new cases) and TB patients who had been previously treated with first- and second-line drugs.

### 2.3 Bacteriological and molecular-genetic methods

The biological materials included sputum, bronchoalveolar lavage fluid, and lung tissues. Each sample was examined using microscopy, culture techniques, and molecular genetic tests.

DNA extraction and real-time PCR for the genetic markers of *M. tuberculosis complex* were carried out with the Amplitub-RV kit (Syntol, Russia), following the manufacturer's instructions. If the DNA from *M. tuberculosis* was detected in the clinical sample in sufficient quantity to identify mutations associated with drug resistance, the sample was further examined using the TB-TEST (BIOCHIP-IMB, Russia), according to the manufacturer's instructions. The assay is based on the amplification of 17 fragments of the *M. tuberculosis* genome using the universal primer adapter technique and heat pulses at the elongation step, followed by hybridization on a microarray. The TB-TEST determines the genotypes of *M. tuberculosis* that are endemic to the Russian Federation. These genotypes include Beijing, Beijing B0/W148, LAM, Haarlem, and Ural, among others. This TB-TEST also detects 116 mutations carrying drug resistance in the following genes: *rpoB, katG, inhA, ahpC, gyrA, gyrB, rrs, eis*, and *embB*.

In 2015, the World Health Organization (WHO) recognized the UNIIF-NMRC as a National Centre of Excellence for the WHO TB Supranational Reference Laboratory Network. Since 2017, this laboratory has taken part in national and international quality assurance programs for testing TB drug susceptibility using the TB-TEST.

The diagnostic material was inoculated into Löwenstein–Jensen and Mycobacteria Growth Indicator Tube (MGIT) media following the Russian clinical recommendations (20). If drug-resistance mutations were not obtained directly from the biological materials, a culture was used.

We only analyzed the results of the phenotypic drug-susceptibility test (DST) in cases where an obtained *M. tuberculosis* culture had the mutation status of “Associated with R–Interim” or “Uncertain significance,” according to the *Catalog of Mutations in Mycobacterium Tuberculosis Complex* (24). During 2017–2020, our laboratory employed the absolute concentration method for drug susceptibility testing using the Löwenstein–Jensen medium, Sensititre Mycobacterium tuberculosis MYCOTBI AST Plate, and BACTEC MGIT system.

### 2.4 Variables and data collection

The database with drug-resistance mutations and genotypes also included patient information on gender, age, diagnosis, region of residence, comorbidities (HIV and hepatitis), vulnerable groups, treatment duration and treatment regimen, and sample types (respiratory or surgical). The data sources included clinical records that were extracted and entered into a spreadsheet. The database was accessed between June 2022 and March 2023.

### 2.5 Analysis

We defined genetic patterns based on a combination of *M. tuberculosis* genotypes and drug-resistance mutations and counted the number of patients who were infected by *M. tuberculosis* with the same combinations (patterns). We assumed that *M. tuberculosis* with unique patterns represented minimal transmission, and the more patients with the same *M. tuberculosis* pattern, the more that transmission had probably taken place, hence biologically successful variants. We analyzed the respiratory and surgical samples separately, considering the tuberculosis treatment duration. Cross-tabulation was done, and the data were analyzed using a chi-square test. Differences were considered significant at *p* < 0.01.

### 2.6 Ethical approval

This study was approved by the Local Ethics Committee of the National Medical Research Center for Phthisiopulmonology and Infection Disease, Ekaterinburg, Russia (approval protocol No 102, 24.11.2021).

## 3 Results

From January 2017 to December 2020, a total of 2,735 TB patients were examined in the UNIIF-NMRC clinic, and 748 had positive DNA results for *M. tuberculosis* from clinical samples and/or culture. Of these patients, we only included patients from Sverdlovsk Oblast who had bacterial mutations conferring resistance to rifampicin and isoniazid, which accounted in 345 patients; of these 345 patients, 193 (55.9%) had culture results available.

### 3.1 General characteristics of patients

Table 1 shows the clinical and demographic characteristics of the patients. Of all the patients, 67.5% were men, and the median age was 37 years. There were 62 patients (17.9%) with HIV/TB co-infection and 82 (23.8%) with hepatitis; 76 (22.0%) of them were former prisoners; 62 (17.9%) patients were Yekaterinburg citizens, while the others were from different places in Sverdlovsk Oblast. In total, 142 (41.2%) DNA samples were isolated from the sputum or bronchoalveolar lavage fluid samples collected from patients before TB treatment and during treatment with second-line TB drugs for varying durations; 125 (88.0%) respiratory samples were culture-positive. In total, 203 (58.8%) DNA samples were obtained from the biological material, of which 142 (69.9%) had *M. tuberculosis*–positive sputum before surgery and had different durations of TB treatment with second-line TB drugs; only 68 (33.5%) surgical samples were culture-positive. The remaining 61 (30.1%) samples were *M. tuberculosis*–negative before surgery, and these patients neither received treatment nor were treated with first- or second-line drugs before surgery.

**Table 1:** Patterns of pulmonary MDR tuberculosis by patient and M. tuberculosis characteristics in Sverdlovsk oblast, Russia, 2017–2020.

**Characteristics**		**Unique patterns *N* = 127, *n* (%)**	**Patterns with 2–6 patients (Group 2) *N* = 107, *n* (%)**	**Patterns with 7–18 patients (Group 3) *N* = 111, *n* (%)**
**Patient characteristics**				
Men		86 (67.7)	77 (71.9)	70 (63.1)
Median age		38	36	36
Yekaterinburg citizen		22 (17.3)	17 (15.9)	23 (20.7)
HIV-positive		24 (18.9)	19 (17.8)	19 (17.1)
Hepatitis-positive		28 (22.0)	26 (24.3)	28 (25.2)
Former prisoner		26 (20.5)	29 (27.1)	21 (18.9)
DNA obtained from respiratory sample		65 (51.2)^*^	47 (43.9)^*^	30 (27.0)^*^
before treatment		7 (5.5)	11 (10.3)	10 (9.0)
up to 12 months/SLDs		20 (15.7)	16 (14.9)	11 (9.9)
more than 12 months/SLDs		38 (29.9)^*^	20 (18.7)^*^	9 (8.1)^*^
DNA obtained from surgical material		62 (48.8)^*^	60 (56.1)^*^	81 (72.9)^*^
*M. tb* “+” before surgery	up to 12 months/SLDs	23 (18.1)	28 (26.2)	25 (22.5)
	more than 12 months/SLDs	19 (14.9)^*^	15 (14.0)^*^	32 (28.8)^*^
M. tb “–” before surgery	before treatment	4 (3.1)	1 (0.9)	4 (3.6)
	FLDs	13 (10.2)	12 (11.2)	17 (15.3)
	SLDs	3 (2.4)	4 (3.7)	3 (2.7)
***M. tuberculosis*** **characteristics**				
**Genotype**				
Beijing B0/W148		75 (59.1)^*^	71 (66.4)^*^	104 (93.7)^*^
Other Beijing types		39 (30.7)^*^	32 (29.9)^*^	7 (6.3)^*^
Non-Beijing types		13 (10.2)^*^	4 (3.7)^*^	0^*^
**Resistance mutations**				
*gyrA/B* genes (fluoroquinolones)		93 (73.2)^*^	47 (43.9)^*^	34 (30.6)^*^
*rrs/eis* genes (aminoglycosides)		83 (65.4)	67 (62.6)	57 (51.4)
*embB* gene (ethambutol)		105 (82.7)	81 (75.7)	86 (77.5)
**Year of registration**				
2017		25 (33.3)	21 (28.0)	29 (38.7)
2018		34 (34.0)	32 (32.0)	34 (34.0)
2019		40 (39.2)	32 (31.3)	30 (29.4)
2020		28 (41.2)	22 (32.3)	18 (26.5)

SLDs, second-line drugs; FLDs, first-line drugs; *M. tb, Mycobacterium tuberculosis*. ^*^Statistically significant at the *p* < 0.01 level.

### 3.2 Genotype and drug-resistant mutations

**Figure 1:**
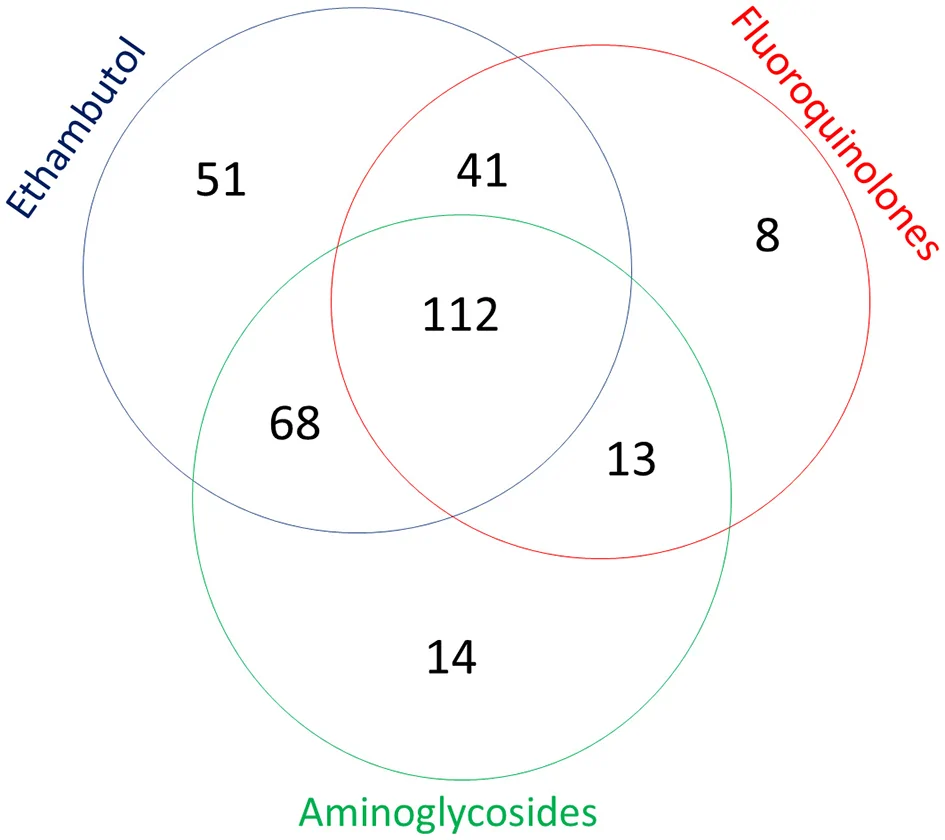
Combination of mutations in genes associated with resistance to fluoroquinolones, aminoglycosides, and ethambutol of 345 *Mycobacterium tuberculosis* cases detected in Sverdlovsk Oblast, 2017–2020.

Table 1 also shows the number of patients infected by *M. tuberculosis* belonging to various genotypes and mutations in three pattern groups. Figure 1 illustrates the combination of mutations in the *gyrA/B* genes (FQs), *rrs/eis* genes (aminoglycosides), and *embB* gene (ethambutol) for all *M. tuberculosis* cases. The Beijing genotype was found in 328 (95.1%) cases and, of these, 250 (72.4%) belonged to the Beijing B0/W148 cluster. In total, 272 cases (78.8%) of *M. tuberculosis* had mutations in the *embB* gene, 207 cases (60.0%) had mutations in the *rrs/eis* genes, and 174 cases (50.4%) had mutations in the *gyrA/B* genes.

Table 2 shows the types of drug-resistant mutations by genotype. The majority of identified mutations were within hotspot codons. The most frequent mutation to rifampicin was *rpoB*S531L, and it was present in 306 (88.7%) samples. For isoniazid resistance, the mutation *katG*S315T(1) was identified in 311 (90.1%) samples. In one case, mutation *katG* I335V with uncertain significance was detected, but a clinical isolate was not obtained. There were 29 different types of mutations and their combinations in *gyrA* and/or *gyrB* genes; the most common mutations were *M. tuberculosis* with *gyrA*D94G plus *gyrA*S95T mutations (61; 35.1%) and *gyrA*A90V (46; 26.4%). There were four samples with *gyrA*G88A mutation (uncertain significance), according to phenotypic DST. All isolates were found to be resistant to ofloxacin (2 mg/L) using the absolute concentration method. Additionally, two isolates were found to be resistant to moxifloxacin (0.25 mg/L) using the Bactec MGIT system, while resistance testing was not conducted for the other two isolates.

**Table 2:** Mutations in genes associated with drug resistance in *M. tuberculosis* by genotype, in Sverdlovsk Oblast, 2016–2020.

**Genotype**	**Beijing B0/W148**	**Other Beijing types**	**Non-Beijing types**	**Total**
**Number of patterns**	107 (61.5%)	52(29.9%)	15 (8.6%)	**174**
**Type of mutation to rifampicin (** ***rpoB*** **)**				
S531L	240	54	12	**306**
S531L, L533P	2	0	0	**2**
H526N	5	7	1	**13**
H526L	1	5	0	**6**
D516Y	0	5	0	**5**
D516V	0	1	3	**4**
L533P	2	1	1	**4**
D516G, L511P	0	2	0	**2**
H526Y	0	1	0	**1**
N513L	0	1	0	**1**
L511P	0	1	0	**1**
**Total** ***rpoB***	**250**	**78**	**17**	**345**
**Type of mutation to isoniazid (** ***katG/inhA*** **)**				
S315T(1)	241	63	7	311
315T(1)/c15t	4	10	10	24
S315T(1)/t8a	4	0	0	4
S315T(1)/t8g	1	0	0	1
S315T(1)/I335V	0	1	0	1
I335V^*^	0	1	0	1
-/c15t	0	3	0	3
**Total** ***katG/inhA***	**250**	**78**	**17**	**345**
**Type of mutation to fluoroquinolones** (***gyrA/gyrB***)				
D94G, S95T	46	12	3	**61**
A90V	36	9	1	**46**
D94A, S95T	14	3	0	**17**
S91P	6	1	0	**7**
D94N	4	1	1	**6**
D94N, S95T	2	2	1	**5**
G88A^**^	3	0	1	**4**
Other mutations (21 types: G88C, A90V+D94G, D94A, D94G, D94Y, A90V+S91P, A90V+N538D, A90V+D94G+S95T, A74S^*^+A94G+S95T, D94Y+S95T, D94H+S95T, P102H^*^, D94A+S95T+*A5*4*3V*, D94G+S95T+P102H, D94N+S95T+T539N, *R4*85C^*^, *S4*86F^**^, *I5*25L^*^, *D5*00N^**^, *E5*4*0D*, *T5*39N^*^)	19	8	1	**28**
**Total** ***gyrA/B***	**130**	**36**	**8**	**174**
**Type of mutation to ethambutol (** ***embB*** **)**				
M306V	128	26	2	**156**
Q497R	32	7	1	**40**
G406A	16	4	0	**20**
Q497K	12	1	1	**14**
M306I(1)	6	2	5	**13**
R354A	4	6	1	**11**
G406S	2	6	0	**8**
M306I(2)	0	4	1	**5**
N296H^*^	2	0	0	**2**
G406D	1	1	0	**2**
Y319S	0	1	0	**1**
**Total** ***embB***	**203**	**58**	**11**	**272**
**Type of mutation to aminoglicozides (** ***rrs/eis*** **)**				
–*/*g10a	74	5	1	**80**
a1401g	66	6	5	**77**
***–**/*c14t	12	15	0	**27**
–*/*g37t^*^	6	6	0	**12**
–*/*c12t	6	2	2	**10**
g1484t/g10a	1	0	0	**1**
**Total** ***rrs/eis***	**165**	**34**	**8**	**207**

The TB-TEST determines the genotypes of *M. tuberculosis* endemic to the Russian Federation: Beijing B0/W148, Beijing (Others Beijing types), LAM, Haarlem, Ural and other (Non-Beijing types), and 116 mutations carrying drug resistance: in the ***rpoB*** (*del507, L511P/R, S512T/R, N513L/K, N513G, D516Y/G/V/E/A, S522L, H526Y/F/C/D/N/L/P/R/Q, S531L/C/W/N, L533P*), ***katG*** (*S315T(1)/I/N/R(1)/G/T(2)/R(2), W328G/L/C, I335V*), ***inhA*** (*A8, G8, T15, G16, T24*), ***ahpC*** (*A6, T10, A10, A9, T12*), ***gyrA*** (*H70R, A74S, T80A, G88A/C, A90V/G, S91P, D94H/A/N/G/Y/V, S95T, P102H*), ***gyrB*** (*R485C/H/L, S486F, D500H/N/A, G509C/A, I525L, D533A, N538D/Y/K/T, T539I/N/P, E540D/D-2/V, A543T/V*), ***rrs*** (*a1401g, C1402t/a, g1484t*), ***eis*** (*c14t, a13g, c12t, g10a/c, g37t*), and ***embB*** (N296H, S297A, M30V/L/I1/I2/I3, V309F, A313V, Y319C/S/D, D328Y/G, D354A, E378A, G406A/S/D/C, Q497R/K/P) genes. ^*^Mutations with “uncertain significance,” ^**^Mutations “associated with R–Interim,” according to the *Catalog of Mutations in Mycobacterium Tuberculosis Complex* (24).

In six cases, mutations in the *gyrB* gene were detected. Clinical isolates were not obtained for two patients with *M. tuberculosis* containing *gyrB* mutations: R485C (uncertain significance) and S486F (associated with R–Interim). Clinical isolates with resistance to ofloxacin (2 mg/L) (absolute concentration method) and moxifloxacin (0.25 mg/L) using the BACTEC MGIT system were obtained for patients with *M. tuberculosis gyrB* mutations: T539N and I525L (uncertain significance) and D500N (associated with R–Interim). There were 11 different mutations in the *embB* gene associated with ethambutol resistance, and M306V was present in 156 (57.3%) samples. For two cases with *M. tuberculosis embB* mutation N296H, a clinical isolate was obtained from one case, and it was resistant to ethambutol (MIC 8 mg/L on Sensititre *Mycobacterium tuberculosis* MYCOTBI AST Plate). For resistance to aminoglycosides, two mutations were dominant: *rrs* a1401g, found in 77 (37.2%) samples, and *eis* g10a, found in 80 (38.6%) samples. Of the 12 *M. tuberculosis* samples with *eis* g37t mutation (uncertain significance), clinical isolates were obtained for 8 samples, and 7 of these isolates were resistant to kanamycin (30 mg/L, absolute concentration method).

### 3.3 Genetic patterns of MDR *M. tuberculosis*

In total, 174 different genetic patterns (a combination of *M. tuberculosis* genotypes and drug resistance mutations) were obtained from the 345 *M. tuberculosis* samples. We divided the patients into three groups: Group 1 included 127 patients with unique *M. tuberculosis* genetic patterns; Group 2 consisted of 107 patients with low-frequency patterns (37 patterns), where each pattern had 2–6 patients (21 sets of 2 patients, 7 sets of 3 patients, 4 sets of 4 patients, 2 sets of 5 patients, and 3 sets of 6 patients); and Group 3 comprised of 111 patients with high-frequency genetic patterns (10 patterns) that had more than 7 patients. The categorization into Group 2 and Group 3 was made conditionally so that the groups would be approximately the same size, allowing us to compare the impact of genetic diversity.

The proportions of patients belonging to the three groups of patterns are presented in Table 1. The proportion of Beijing B0/W148 in Group 3 (93.7%) was significantly higher than in Group 2 (66.7%) and Group 1 (59.1%). The proportion of *M. tuberculosis* with mutations in *gyrA/B* genes was significantly higher in Group 1 (73.2%) than in Group 2 (43.9%) and Group 3 (30.6%). The proportion of patients with *M. tuberculosis* obtained from respiratory material was significantly higher in Group 1 (51.2%) than in Group 2 (43.9%) and Group 3 (27.0%). For surgical samples, the proportions of patients were 48.8% in Group 1, 56.1% in Group 2, and 72.9% in Group 3.

An analysis of patterns across different categories of patients revealed that unique patterns were significantly more prevalent in patients with *M. tuberculosis*–positive sputum and a treatment duration exceeding 12 months (median: 44 months; 29.9%; Table 1). This patient group also exhibited a higher proportion of mutations in *gyrA/B* genes (91.0%) and included HIV-positive patients (43.3%), hepatitis B/C–positive patients (44.8%), and former prisoners (50.7%; Table 3).

**Table 3:** Characteristics of patients with multidrug-resistant pulmonary tuberculosis and their examined surgical material in Sverdlovsk Oblast, 2017–2020.

Characteristics of material		**Total**	**Category of patient**			**Mutations in *gyrA/B* genes**
			**HIV positive**	**Hepatitis positive (C/B)**	**Former prisoners**	
DNA from respiratory sample		142	36 (25.3)	42(29.5)	63(44.5)	106(74.6)
Before treatment		28	2(7.1)	2(7.1)	23(10.7)	14 (50.0)
Up to 12 months/SLDs		47	5(10.6)	8 (17.0)	6(12.7)	31(65.9)
More than 12 months/SLDs		67	29(43.3)	32 (44.8)	34(50.7)	61(91.0)
DNA from surgical material		203	26(12.8)	40(19.7)	33(16.3)	68(33.5)
*M. tb* “+” before surgery	Up to 12 months/SLDs	76	7 (9.2)	13(17.1)	8(10.5)	27(35.5)
	More than 12 months/SLDs	66	14 (21.2)	17 (25.7)	16(24.2)	24(36.4)
*M. tb* “–” before surgery	Before treatment	9	1(11.1)	1 (11.1)	0	3(33.3)
	FLDs	42	4 (9.5)	8 (19.0)	8(19.0)	10(23.8)
	SLDs	10	0	1 (10.0)	1(10.0)	4 (40.0)

SLDs, second-line drugs; FLDs, first-line drugs; *M. tb, Mycobacterium tuberculosis*.

In patients with *M. tuberculosis*–positive sputum, the proportion of mutations in *gyrA/B* genes depended on the duration of treatment: before treatment (50.0%), up to 12 months (65.9%), and more than 12 months (91.0%; Table 3). The three groups of patterns were not significantly different in the other variables.

In Table 4, the genetic patterns in Group 3 (patterns with more than seven patients) are listed. All patterns except one belonged to the Beijing B0/W148 genotype and had the same mutations *katG* S315T1 and *rpoB* S531L. The variability was due to the presence or absence of mutations in *embB* (M306V or Q497R), *gyrA (*D94G+S95T or A90V*), rrs* (a1401), and *eis* (g10a) genes and, for each drug, only two types of mutations were noted. The proportion of categories of patients (new cases, duration of treatment, HIV/hepatitis, and prisoners) varied among the patterns. In general, the duration of tuberculosis treatment before obtaining samples was longer in cases in which mutations in *gyrA/B* genes were present (Group 3 patterns). However, due to the small number of samples in each pattern, no statistically significant differences were identified.

**Table 4:** Mutations and genotypes of the most common genetic patterns (Group 3) of *Mycobacterium tuberculosis* and clinical characteristics of patients, in Sverdlovsk Oblast, 2017–2020.

**Fluoroquinolones (*gyrA*)**	**Aminoglycosides (*rrs*, *eis*)**	**Etambutol (*embB*)**	**Genotype**	**New TB**	**Duration of treatment, month median**	**Former prisoners**	**HIV positive**	**Hepatites positive**	**Year of isolation**				**Total number of patients with pattern**
									**2017** ***N*** = **75**	**2018** ***N*** = **100**	**2019** ***N*** = **102**	**2020** ***N*** = **68**	
wt	wt	wt	Beijing	0	6	2(28.5)	2(28.5)	1(14.2)	1	3	2	1	**7**
wt	wt	wt	Beijing B0/W148	4	10	2(11.1)	1(5.6)	2(11.1)	4	4	9	1	18
wt	wt	Q497R	Beijing B0/W148	2	12	2(18.2)	0	1(9.1)	3	4	1	3	11
wt	wt	M306V	Beijing B0/W148	3	9.5	2(18.2)	1(9.1)	3(27.3)	5	2	4	0	11
D94G S95T	wt	M306V	Beijing B0/W148	0	22	1(14.3)	2(28.6)	1(14.2)	3	2	1	1	7
wt	g10a	M306V	Beijing B0/W148	2	10	6(35.3)	3(17.6)	5(29.4)	3	7	3	4	17
D94G S95T	g10a	M306V	Beijing B0/W148	0	8	1(11.1)	3(33.3)	4(44.4)	3	2	0	4	9
wt	a1401g	M306V	Beijing B0/W148	1	13	1(7.7)	1(7.7)	3(23.1)	3	5	3	2	13
D94G S95T	a1401g	M306V	Beijing B0/W148	1	20	2(18.2)	5(45.5)	7(63.6)	2	2	5	2	11
A90V	a1401g	M306V	Beijing B0/W148	1	25	2(28.6)	1(14.3)	1(14.2)	2	3	2	0	7

All *M. tuberculosis* had *rpob* S531L and *katG* S315T mutation.

## 4 Discussion

In this study, we identified 174 distinct *M. tuberculosis* genetic patterns among 345 patients with MDR-TB. Of these, 36.8% were unique, 31.0% shared the same pattern with 2–6 others, and 32.2% shared the same pattern with 7–18 others. The Beijing B0/W148 genotype was more frequent in the group with 7–18 identical combinations; unique patterns were significantly more common in the patient group with *M. tuberculosis*–positive respiratory samples and a treatment duration exceeding 12 months. This group also exhibited a higher proportion of mutations in *gyrA/B* genes and included HIV-positive patients, hepatitis (B/C)–positive patients, and former prisoners.

These observations suggest that patients with “unique” *M. tuberculosis* patterns have poorer treatment adherence and may have been the primary source of the new pathogen variants, mainly due to the emergence of mutations in *gyrA/B* genes. The emergence of FQ resistance (often with a wide range of mutations and a larger number of unique profiles) may lead to less effective chemotherapy (or may result from inadequate chemotherapy), consequently resulting in ongoing bacterial excretion. The pathogen's biological traits (fitness) or the brief period after their emergence may have contributed to our observation that the variants were identified as unique.

The apparent increase in FQ resistance may also be related to the quality of laboratory diagnostics. Until 2014, in Sverdlovsk Oblast, identifying FQ susceptibility was based on ofloxacin using the absolute concentration method. Molecular genetic testing, since 2014, and the BACTEC MGIT, since 2018, determined mutations to FQ and susceptibility to levofloxacin and moxifloxacin, respectively. The WHO, in 2018, and Russian clinical recommendations, in 2022, lowered the critical concentration for BACTEC MGIT from 1.5 mg/L to 1 mg/L for levofloxacin and from 0.5 mg/L to 0.25 mg/L for moxifloxacin. This change suggests that, in previous years, we may have underdiagnosed real FQ resistance based on phenotypic tests, leading to inadequate TB treatment and a potential increase in TB drug resistance. These factors may account for the wide range of unique genetic patterns associated with different mutation types in *gyrA/B* genes, which we detected in our study.

Our data confirm that several factors are associated with the high levels of MDR/pre-XDR TB in Sverdlovsk Oblast. One significant factor is the dominance of the Beijing genotype (in particular, Beijing B0/W148, which was more frequent in the high-frequency pattern group). This strain has been circulating in Sverdlovsk Oblast (10, 11) and other regions inside and outside of Russia (7–9, 12, 14) for several decades. It is known that a higher mutation rate accounts for the higher adaptation ability and drug-resistance rates of the Beijing lineage (25, 26). The second factor is the high prevalence of HIV infection in the Sverdlovsk region, which may facilitate the circulation of drug-resistant mycobacteria with reduced fitness, allowing for further evolution. The relatively low treatment success rate of MDR-TB patients (62% in 2020; (22)) indicates that *M. tuberculosis* has the opportunity to evolve and acquire new mechanisms of drug resistance.

Upon analyzing the trend in the proportion of *M. tuberculosis* belonging to different pattern groups, we observed that over the 4-year period, the proportion of *M. tuberculosis* patterns gradually increased from 33.3% to 41.2%, while the proportion of *M. tuberculosis* with dominant patterns gradually decreased from 38.7% to 26.5%. This may be because new mutations accumulate faster than the spread of the pathogen's successful variants; however, a longer observation period is necessary to establish statistical significance.

The studies from different regions of Russia and beyond indicate that FQ resistance is significantly more likely to be acquired than other resistance genotypes, suggesting that *gyrAB* mutants may have impaired transmission fitness, which impedes the spread of MDR/FQ resistant clones (11, 27–29). An analysis of whole-genome sequencing data regarding Beijing B0/W148 isolates revealed that the gradual accumulation of resistance mutations is consistently negatively associated with epidemiological success; however, this association can be weakened by compensatory mutations in XDR isolates, which can neutralize the negative impact of resistance mutations on mycobacterial fitness (14). In this study, we identified several dominant patterns (combined 7–18 patients), including those involving mutations in *gyrA/B* genes (mainly *gyrA* D94G S95T). These patterns were observed across all patient groups, suggesting their potential for successful transmission. This finding warrants further investigation.

A limitation of the study was the insufficient discriminative power of the TB-TEST for genotyping, which only allowed categorization based on the detection of single nucleotide polymorphisms in some specific loci. As a result, we could not identify clusters that require MIRU-VNTR (mycobacterial interspersed repetitive units, variable number of tandem repeats) or whole-genome sequencing, and we lacked information about epidemiological links. However, the TB-TEST is useful for monitoring trends in genotype frequency and resistance mutation patterns, especially in cases when *M. tuberculosis* isolates are not obtained, and for identifying groups that need special follow-up.

Considering that the clinical isolates of *M. tuberculosis* were obtained from only 55.9% of patients, we performed phenotypic drug susceptibility tests only in cases with positive culture results and when mutations categorized as “uncertain significance” or “associated with R–Interim,” according to the *Catalog of Mutations in Mycobacterium Tuberculosis Complex* (24), were detected. We observed a strong correlation between the results of the TB-TEST and the phenotypic methods in these cases.

We included all patients hospitalized in the UNIIF-NMRC clinic with pulmonary MDR-TB during a 4-year study period (2017–2020), who had TB-TEST results; however, estimates suggest that this sample size represented only approximately 5% of all MDR-TB cases in Sverdlovsk Oblast, indicating that it was a highly selected group. Therefore, the proportion of patients with unique strains is likely higher than the rest of the MDR cases in the oblast. However, within our selected group of patients, we found different transmission potentials in our sample groups, which is of great interest from a public health perspective. However, the patients included in our study were a selected group of “problem cases,” and we cannot be certain that they represent the whole oblast or all MDR-TB cases, especially new TB cases. We likely overlooked TB patients with advanced HIV, as those with CD4 count of <400 cells/ml were not admitted to the UNIIF hospital.

Monitoring drug resistance in TB is an important aspect of evaluating a TB program. This study offers new insights into categorizing TB patients based on the patterns of *M. tuberculosis* genotypes and resistance mutations using a standard microarray assay TB-TEST as a screening method. Given the high prevalence of MDR-TB in Sverdlovsk Oblast, these data allow us to identify the most common patterns, suggesting the biological success of the TB pathogen and its transmission.

## Data Availability

The raw data supporting the conclusions of this article will be made available by the authors, without undue reservation.
